# Current and future perspectives in the treatment of multidrug-resistant Gram-negative infections

**DOI:** 10.1093/jac/dkab352

**Published:** 2021-11-21

**Authors:** Matteo Bassetti, Javier Garau

**Affiliations:** 1 Clinica Malattie Infettive, Ospedale Policlinico San Martino—IRCCS, Genoa, Italy; 2 Department of Health Sciences, University of Genoa, Genoa, Italy; 3 Hospital Universitari Mutua de Terrassa, Barcelona, Spain; 4 Clínica Rotger Quironsalud, Palma de Mallorca, Spain

## Abstract

Microbial resistance is a serious threat to human health worldwide. Among the World Health Organisation’s list of priority resistant bacteria, three are listed as critical—the highest level of concern—and all three are Gram-negative. Gram-negative resistance has spread worldwide via a variety of mechanisms, the most problematic being via AmpC enzymes, extended-spectrum β-lactamases, and carbapenemases. A combination of older drugs, many with high levels of toxicity, and newer agents are being used to combat multidrug resistance, with varying degrees of success. This review discusses the current treatments for multidrug-resistant Gram-negative bacteria, including new agents, older compounds, and new combinations of both, and some new treatment targets that are currently under investigation.

## Introduction

Antimicrobial resistance is a complex and dynamic phenomenon, mostly relying on a complicated interaction between direct factors, such as misuse of antimicrobials in humans and agricultural animals, indirect factors, such as environmental pollution and poor sanitation, and the innate characteristics of the bacteria themselves.^1^ Previous antibiotic exposure, underlying diseases, and invasive procedures have been identified by some researchers as the risk factors most associated with resistance.^2^ However, the risk factors for spread of resistance vary by geography: according to the WHO, antimicrobial resistance in developing countries is more likely to be spread through poor sanitation and lack of clean drinking water,^3^ whereas data from the United States (US) indicate that one in five resistant infections are caused by exposure to contaminated food or animals.^4^ In Europe, factors for spread of antimicrobial resistance have been cited as cross-border transfer of patients carrying MDR bacteria, transmission of MDR pathogens in and between healthcare settings, antimicrobial over-use and misuse, and inconsistent infection control practices.^5^ The Asia-Pacific region, home to two-thirds of the world’s population, is highly vulnerable to increased antimicrobial resistance. Here, the spread of resistance is more likely driven by factors such as rapidly growing and densely populated cities and increasing wealth and the associated increase in mass-farming practices.^6^ Clearly, detailed information on the relative contribution of the various factors to the overall global problem of MDR infections has not been adequately researched and is yet to be fully elucidated.^1^ There is a need, therefore, to address the multiple factors associated with MDR infections both across the globe and locally based on the different epidemiological and societal scenarios.

The specific mechanisms by which pathogens become resistant to antimicrobials may be innate, adaptive or acquired by the organism, and include mechanisms that limit drug penetration into, or increase drug removal from, the bacteria, modification of the drug targets through mutation selection, or enzymatic inactivation of drugs. Regardless of the mechanism, antimicrobial resistance is already limiting our ability to successfully treat infections,^7^ and thus poses a serious threat to human health.^8^ MDR Gram-negative organisms, particularly carbapenem-resistant Enterobacterales (formerly known as Enterobacteriaceae), carbapenem-resistant *Pseudomonas aeruginosa*, and extensively-drug-resistant (XDR) *Acinetobacter baumannii*, present a particularly grave threat worldwide.^9^

This review discusses the current and future burden of MDR Gram-negative infections, treatment options—existing and potential—and other considerations in the overall management of MDR Gram-negative infections, including the importance of understanding local epidemiology and enabling rapid diagnosis.

## The current and future burden of MDR Gram-negative infections

The increased threat from Gram-negative MDR species is widely acknowledged by global and national organizations including the WHO,^8^ European Centre for Disease Prevention and Control,^10^ Infectious Diseases Society of America (IDSA),^4^ and the US CDC.^11^ Indeed, among the WHO’s list of priority resistant bacteria for 2016–17, three are described as critical—the highest level of concern—and all three are Gram-negative, namely carbapenem-resistant Enterobacterales, carbapenem-resistant *A. baumannii*, and carbapenem-resistant *P. aeruginosa.*^8^ According the 2013 CDC report, 6.6% of the 140 000 most serious healthcare-related Enterobacterales infections occurring annually in the US are resistant to carbapenems, while 63% of the 12 000 *Acinetobacter* infections, and 13% of the estimated 51 000 *Pseudomonas* infections are multidrug resistant.^12^ While the 2019 report describes a relatively reduced incidence of many of these infections, the incidence of carbapenem-resistant infections has remained stable, and MDR organisms are still considered a global critical threat.^11^ In Europe, the highest levels of MDR infections were reported for *P. aeruginosa*,^13^ with carbapenem resistance in 2017 reportedly as high as 63% in some countries in Southern and South Eastern Europe.^14^

In a 2016 analysis of 175 studies conducted in several countries in Southeast Asia, carbapenem-resistant Enterobacterales rates were relatively low (2.8%), while carbapenem-resistant *A. baumannii* and carbapenem-resistant *P. aeruginosa* rates were 73.0% and 29.8%, respectively; however, the prevalence of all three species of resistant bacteria was rising.^15^ In China, data from the China Antimicrobial Surveillance Network showed that 71.4% of *Acinetobacter* spp. strains, 10% of Enterobacterales strains and 20%–30% of *P. aeruginosa* strains isolated in 2017 were resistant to carbapenems.^16^

The most serious outcomes of Gram-negative MDR occur in critically ill and other high-risk patients, and MDR is associated with high levels of mortality and inappropriate use of antibacterial treatment in patients with MDR infections. For example, in neutropenic patients, carbapenem resistance is increasing, particularly among *Pseudomonas* species, and mortality rates for neutropenic patients (primarily those with haematological malignancies) with carbapenem-resistant bloodstream infections (BSI) range from 33.3% to 71.4%.^17^ In haematopoietic stem cell recipients, inappropriate empirical antibacterial therapy was reportedly given in 46.2% of cases of MDR Gram-negative infection.^18^

## Mechanisms of resistance

Broadly, organisms develop resistance to multiple antimicrobials via successive mutations, dissemination of multiresistance plasmids or transposons, or a combination of both processes.^19^

Specific mechanisms of resistance developed by organisms are more complex. Among the most problematic and relevant resistance mechanism developed by Gram-negative bacteria is that of β-lactamases, enzymes that transfer resistance to β-lactam (BL) antibiotics, a broad range of highly useful compounds that includes penicillin derivatives, cephalosporins, monobactams, and carbapenems. There are two main classification systems for β-lactamases: the Ambler classification system in which enzymes are classified according to their protein sequences (Ambler classes A, B, C and D; Table 1)^20^ and the Bush–Jacoby system, which classifies the enzymes according to their clinical phenotypes.^21^

**Table 1.** Ambler classification of β-lactamases by main antibacterial substrate^21^

β-Lactamase enzyme	Main antibacterial substrate	Inhibited by
Ambler class A		
PC 1	Penicillins	Clavulanic acid or tazobactam
TEM-1, TEM-2, SHV-1	Penicillins, early cephalosporins	Clavulanic acid or tazobactam
TEM-3, SHV-2, CTX-M-15, PER-1, VEB-1	Extended-spectrum cephalosporins, monobactams	Clavulanic acid or tazobactam
TEM-30, SHV-10	Penicillins	
TEM-50	Extended-spectrum cephalosporins, monobactams	
PSE-1, CARB-3	Carbenicillin	Clavulanic acid or tazobactam
RTG-4	Carbenicillin, cefepime	Clavulanic acid or tazobactam
CepA	Extended-spectrum cephalosporins	Clavulanic acid or tazobactam
KPC-2, IMI-1, SME-1	Carbapenems	Clavulanic acid or tazobactam (variable)
Ambler class B		
IMP-1, VIM-1, CcrA, IND-1, L1, CAU-1, GOB-1, FEZ-1	Carbapenems (not monobactams)	EDTA
CphA, Sfh-1	Carbapenems	EDTA
Ambler class C		
* E. coli* AmpC, P99, ACT-1, CMY-2, FOX-1, MIR-1, GC1, CMY-37	Cephalosporins	
Ambler class D		
OXA-1, OXA-10	Cloxacillin	Clavulanic acid or tazobactam (variable)
OXA-11, OXA-15	Extended-spectrum cephalosporins	Clavulanic acid or tazobactam (variable)
OXA-23, OXA-48	Carbapenems	Clavulanic acid or tazobactam (variable)

Carbapenem resistance is particularly serious given that carbapenems are often the last resort in treating infections resistant to other drugs. Carbapenem resistance mechanisms have spread across the world and between organisms, and a wide range of enzymes have been identified among carbapenemase-producing Enterobacterales. These include the serine β-lactamases *Klebsiella pneumoniae* carbapenemase (KPC) (Ambler class A), metallo-β-lactamase (MBL) including New Delhi MBL (NDM) or Verona integron-encoded MBL (VIM), imipenemase (IMP) (Ambler class B) and OXA-48-like carbapenemases (Ambler class D).^22^ KPCs hydrolyse penicillins, cephalosporins, monobactams and carbapenems.^23^^,^^24^ KPC, NDM and OXA-48 enzymes are among the carbapenem resistance mechanisms of greatest concern.^25^

In addition to carbapenemase production, which is a common mechanism of carbapenem resistance in Enterobacterales, other such mechanisms include porin mutations and efflux pump upregulation.^26^ For example, in *P. aeruginosa*, carbapenem resistance occurs as a result of the loss of porin OprD or increased expression of MexAB-OprM, MexXY-OprM or MexCD-Opr efflux pumps, or a combination of the two. In *A. baumannii*, in addition to Ambler class D carbapenemases, such as OXA-23, OXA-40 and OXA-58, carbapenem resistance can result from AdeABC efflux pump overexpression.^26^

Resistance can also be viewed in terms of the specific antibacterials or antibacterial classes that are affected by these mechanisms. Examples include fluoroquinolone resistance, which occurs via mutations in DNA gyrase genes *gyrA and gyrB*; resistance to tigecycline, stemming from mutational upregulation of *arcA/B*-mediated efflux; and resistance to third-generation cephalosporins, which occurs via mutational de-repression of AmpC β-lactamases in certain species, including *Enterobacter* spp.^19^

Conversely, different bacteria also exhibit different levels of resistance to the same antimicrobial. In Canada, nitrofurantoin resistance rates were reportedly 16% in ESBL-producing *E. coli*, 71% in nosocomial ESBL-producing *Klebsiella* spp. and 93% in non-nosocomial ESBL-producing *Klebsiella* spp.^19^

Choice of antibacterial agent also differs between countries, both in terms of empirical therapy and targeted therapy against known pathogens.^27^ For example, a 2017 *post hoc* analysis of the INCREMENT study of treatment of BSI caused by MDR Enterobacterales found that carbapenems are more commonly used as empirical therapy in the USA and Taiwan, while empirical use of a β-lactam + β-lactamase inhibitor (BL/BLI) combination is more widespread in Italy and Turkey. For targeted treatment, regimens comprising a carbapenem plus at least one other agent were used in 17.1% (82/479) of cases overall, and most commonly in Italy (31/115; 27.0%), Greece (16/89; 18.0%) and Turkey (5/27; 18.5%); this despite the high levels of carbapenem resistance in Italy and Greece.^28^ Importantly, therefore, the INCREMENT study found that the differences in antibacterial use in the context of MDR Enterobacterales were not always explained by geographical variations in resistance patterns, and are influenced by historical practices and the clinical presentation and severity of these infections. Overall, however, countries with more carbapenem resistance tend to use more combination therapies.^28^ Robust surveillance systems and high-quality evidence of the efficacy of new treatments are needed to develop antibiotic stewardship protocols in MDR infections, and thereby reduce mortality and morbidity.

Attempts are ongoing to overcome antibacterial resistance by using new agents and combinations of new plus old agents. For example, both old (clavulanic acid, tazobactam) and new (avibactam, vaborbactam, relebactam) BLIs are being used in combination with other agents to counteract β-lactamases, and a number of BL/BLI combinations are now available.^29^^,^^30^ Interestingly, some BLIs also have a BL-enhancing mechanism that is independent of the BLI mechanism.^29^ Another analysis of data from the INCREMENT study showed that BL/BLI combinations with *in vitro* activity were as effective as carbapenems for the empirical or targeted treatment of ESBL Enterobacterales BSI.^31^

## Therapeutic approaches for MDR Gram-negative infections

### The most serious MDR clinical scenarios

**Figure 1.**
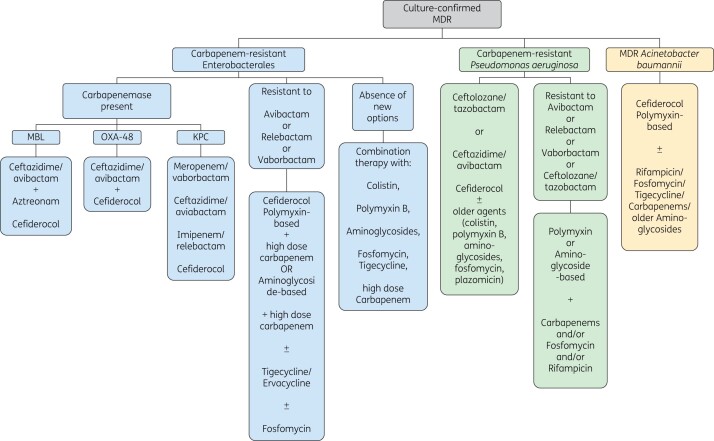
Suggested treatments for carbapenem-resistant Enterobacterales, multidrug-resistant *Pseudomonas aeruginosa*, and multidrug-resistant *Acinetobacter baumannii.*^32^^,^^34^ Treatment choice in each case should also depend on local epidemiology and bacterial susceptibility, and any potential additional toxicity when combining therapy. BL/BLI, β-lactam/β-lactamase inhibitor; KPC, *Klebsiella pneumoniae* carbapenemase; MDR, multidrug-resistant.

The threat of MDR Gram-negative infection is most serious among the critically ill, who often have multiple comorbidities. Recommendations for managing them are organized in one of two ways. Most treatment guidelines for managing MDR Gram-negative infections address broad clinical and epidemiological scenarios, rather than specific MDR Gram-negative pathogens. Furthermore, useful guidelines must address local resistance patterns and accommodate the potential need for rapid changes in recommendations. There are, however, published studies and reviews that contain recommendations presented by MDR pathogen, by antibacterial agent/class, or by disease. Bassetti *et al*.^32^ proposed a treatment algorithm for critically ill patients in the ICU according to MDR pathogen. Broadly, and allowing for local resistance patterns, their first-line recommendations, based on non-clinical and clinical evidence, are: ceftazidime/avibactam (Ambler classes A, C and D), meropenem/vaborbactam (A and C), imipenem/relebactam (A and C), aztreonam/avibactam (A, B and C) or cefiderocol (A, B and D) for carbapenem-resistant Enterobacterales; ceftolozane/tazobactam, imipenem/relebactam (A and C) or cefiderocol (A, B and D) for carbapenem-resistant *P. aeruginosa*; and cefiderocol-based (A, B and D) treatment for carbapenem-resistant *A. baumannii.*^32^^,^^33^ Similarly, a recent review by Peri *et al*.^34^ proposed a set of recommendations specifically for treating carbapenem-resistant Enterobacterales (CRE) and MDR *A. baumannii and P. aeruginosa* (Figure 1). In 2018, Hawkey *et al*.^19^ proposed recommendations for the use of specific antibiotics, but the guidance was not organized by indication. In contrast, the 2020 IDSA guidelines provide indication-specific recommendations for infections caused by different classes of MDR (Tables 2 and 3).^35^

**Table 2.** Antibiotic treatment options recommended by the Infectious Diseases Society of America (IDSA) for ESBL-E and *P. aeruginosa* with difficult-to-treat resistance^35^

Source of infection	ESBL-E		*P. aeruginosa* with difficult-to-treat resistance	
	Preferred treatment	Alternative treatment^a^	Preferred treatment	Alternative treatment^a^
Cystitis	Nitrofurantoin, trimethoprim/sulfamethoxazole.	Amoxicillin/clavulanate, single-dose aminogycosides, fosfomycin (*E. coli* only).	Ceftolozane/tazobactam, ceftazidime/avibactam, imipenem/relebactam, cefiderocol, or a single-dose of an aminoglycoside.	Colistin
Pyelonephritis or cUTI	Ertapenem, meropenem, imipenem/cilastatin, ciprofloxacin, levofloxacin, or trimethoprim/sulfamethoxazole.		Ceftolozane/tazobactam, ceftazidime/avibactam, imipenem/cilastatin/relebactam, and cefiderocol.	Once-daily aminoglycoside.
Infections outside the urinary tract	Meropenem, imipenem- cilastatin, ertapenem Oral step-down therapy to ciprofloxacin, levofloxacin, or trimethoprim/sulfamethoxazole can be considered.^b^		Ceftolozane/tazobactam, ceftazidime/avibactam, or imipenem/cilastatin/relebactam.	Cediferocol Aminoglycoside monotherapy: limited to uncomplicated BSI with complete source control.^c^

This Table is adapted, with permission, from Table 2 in Tamma *et al*.^35^

BSI, blood stream infection; cUTI, complicated urinary tract infection (UTI occurring in association with a structural or functional abnormality of the genitourinary tract, or any UTI in a male patient); ESBL-E, extended-spectrum β-lactamase-producing Enterobacterales.

a If first-line options are not available or tolerated.

b Oral step-down therapy can be considered after (i) susceptibility to the oral agent is demonstrated, (ii) patients are afebrile and haemodynamically stable, (iii) appropriate source control is achieved, and (iv) there are no issues with intestinal absorption.

c Uncomplicated BSIs include a BSI due to a urinary source or a catheter-related BSI with removal of the infected vascular catheter.

**Table 3.** Antibiotic treatment options recommended by the Infectious Diseases Society of America for carbapenem-resistant Enterobacterales^35^

Source of infection	Preferred treatment	Alternative treatment^a^
Cystitis	Ciprofloxacin, levofloxacin, trimethoprim/sulfamethoxazole, nitrofurantoin, or a single-dose of an aminoglycoside. Meropenem^b^ (standard infusion): only if ertapenem resistant, meropenem susceptible, AND carbapenemase testing results are either not available or negative.	Ceftazidime/avibactam, meropenem-vaborbactam, imipenem/cilastatin/ relebactam, and cefiderocol. Colistin (only when no alternative options are available).
Pyelonephritis or cUTI	Ceftazidime/avibactam, meropenem/ vaborbactam, imipenem/cilastatin/ relebactam, and cefiderocol. Meropenem^b^ (extended-infusion): only if ertapenem resistant, meropenem susceptible, AND carbapenemase testing results are either not available or negative.	Once-daily aminoglycosides.
Infections outside the urinary tract if resistant to ertapenem, susceptible to meropenem, AND carbapenemase testing results are either not available or negative.	Meropenem (extended infusion).	Cetazidime/avibactam.
Infections outside the urinary tract if resistant to ertapenem and meropenem, AND carbapenemase testing results are either not available or negative.	Ceftazidime/avibactam, meropenem/ vaborbactam, and imipenem/ cilastatin/relebactam.	Cefiderocol. Tigecycline, eravacycline (intra-abdominal infections).
KPC identified (or carbapenemase positive but identity of carbapenemase is unknown).	Ceftazidime/avibactam, meropenem/ vaborbactam, and imipenem/ cilastatin/relebactam.	Cefiderocol. Tigecycline, eravacycline (intra-abdominal infections).
Metallo-β-lactamase (ie. NDM, VIM or IMP) carbapenemase identified.	Ceftazidime/avibactam + aztreonam, cefiderocol.	Tigecycline, eravacycline (intra-abdominal infections).
OXA-48-like carbapenemase identified.	Ceftazidime/avibactam.	Cefiderocol. Tigecycline, eravacycline (intra-abdominal infections).

This Table is adapted, with permission, from Table 3 in Tamma *et al*.^35^

CRE, carbapenemase-resistant Enterobacterales; cUTI, complicated urinary tract infection (UTI occurring in association with a structural or functional abnormality of the genitourinary tract, or any UTI in a male patient); IMP, imipenemase; KPC, *Klebsiella pneumoniae* carbapenemase; NDM, New Delhi metallo-β-lactamase; VIM, Verona integron-encoded metallo β-lactamases.

a If first-line options are not available or tolerated.

b The vast majority of carbapenemase-producing Enterobacterales infections in the United States are due to bacteria that produce KPC. If a disease-causing Enterobacterales is carbapenemase-producing but the specific carbapenemase enzyme is unknown, it is reasonable to treat as if the strain is a KPC-producer. If a patient is infected with a CRE strain with an unknown carbapenemase status and the patient has recently travelled from an area where metallo-β-lactamases are endemic (e.g. Middle East, South Asia, Mediterranean), treatment with ceftazidime/avibactam plus aztreonam, or cefiderocol monotherapy are recommended. Preferred treatment approaches for infections caused by metallo-β-lactamase producers also provide activity against KPC and OXA-48-like enzymes.

In real-world conditions, several factors prevent the widespread adoption of novel antibiotics, such as higher costs and lack of comparative data versus older drugs, since comparative studies have either not been conducted or were non-inferiority studies.^19^^,^^36–40^ In addition, to be effective in critically ill patients, antibiotic treatment must be administered as early as possible, and conducting antibiotic susceptibility tests can result in delays.^32^ Therefore, specific guidelines for empirical treatment have been developed based on the type of infection. Some of the more serious specific MDR clinical scenarios are discussed here. It is important to point out, however, that the local epidemiological resistance pattern should always be considered.

### Bloodstream infections

BSIs are associated with high morbidity and mortality, with risk factors for MDR BSI including liver disease, diabetes, male sex, age ≥60 years, indwelling catheters, previous therapeutic antimicrobial use and *K. pneumoniae* bacteraemia.^41^^,^^42^ According to Spanish guidelines for managing catheter-related BSI, Gram-negative bacilli are present in 17%–25% of such infections, particularly in patients with special conditions, including spinal cord injuries, femoral catheters, neutropenia and haematological malignancy, or diabetes.^43^ As such, these guidelines recommend empirical antibiotic therapy that includes Gram-negative coverage and must include an anti-pseudomonal agent; however, they do not stipulate how to address resistant organisms.^43^ An Italian surveillance programme demonstrated the rapid increase in carbapenem-resistant *K. pneumoniae* causing BSI, which rose from 1.3% in 2009 to 34.3% in 2013.^44^

In an Italian study of carbapenem-resistant *K. pneumoniae* BSI in critically ill patients (including those with septic shock, chronic renal failure, or neutropenia), one of the factors associated with reduced mortality was receiving an antimicrobial combination that included high-dose meropenem (hazard ratio for death 0.64, 95% CI 0.43–0.95, *P = *0.03).^45^ Another Italian study assessed the efficacy of combination therapy containing high-dose continuous meropenem infusion in which steady-state meropenem concentrations were optimized with therapeutic drug monitoring in patients with KPC-producing *K. pneumoniae* infections, 60% BSIs and 53% meropenem-resistant.^46^ Successful clinical outcomes were achieved in 73% of cases, suggesting that optimizing steady-state meropenem concentrations improves outcomes for KPC-producing *K. pneumoniae* infections with meropenem MIC ≤64 mg/L.

The literature generally appears to support the use of carbapenem-sparing treatment of ESBL BSI, including possible de-escalation to piperacillin/tazobactam or cefepime in non-critically ill patients with BSIs susceptible to these therapies.^47^ However, the international prospective, randomized MERINO study published in 2018 did not establish non-inferiority of piperacillin/tazobactam compared with meropenem for patients with *E. coli* or *K. pneumoniae* BSI and ceftriaxone resistance, with 30 day mortality rates of 12.3% and 3.7%, respectively.^48^ In that study, patients in the carbapenem group were arguably at higher risk, with a higher APACHE II score and prevalence of diabetes.^48^

A survey of 616 infectious disease specialists from 56 countries conducted between 2016 and 2017 showed that BSI management practices vary significantly from institution to institution.^49^ The authors pointed out that such variations pose a threat to antimicrobial stewardship (AMS) programmes, and that evidence-based guidelines for the management of BSIs are urgently needed so that AMS can be implemented effectively at a local level to harmonize treatment.^49^

### Hospital-acquired and ventilator-associated pneumonia

The increase in MDR organisms complicating hospital-acquired pneumonia (HAP) is of great concern. HAP is one of the most common infections in the ICU. According to a recent review of the international literature, there are reportedly as many as 20 cases per 1000 hospital admissions; 44% of all HAP cases are acquired in the ICU with up to 90% requiring ventilation.^7^ European and US data from 2014 showed that *P. aeruginosa* was the Gram-negative pathogen most commonly implicated in HAP and ventilator-associated pneumonia (VAP), accounting for 21% of all cases of HAP in 2014.^50^ Importantly, that study also showed that *P. aeruginosa* had reduced susceptibility to most antimicrobials tested, including ceftazidime (68.7%/79.6% susceptibility in Europe/US), meropenem (65.8%/76.3%), and piperacillin/tazobactam (63.9%/72.9%).^50^

The 2016 IDSA guidelines for managing HAP/VAP strongly recommend the use of individual hospital antibiograms to reduce patient exposure to unnecessary antibiotics and reduce the development of antibiotic resistance, and in particular to reduce the use of dual Gram-negative and empirical MRSA antibiotic treatment.^51–53^ Treatment depends largely on the causative MDR pathogen. Watkins *et al*.^54^ recommended the use of carbapenems first line in HAP caused by ESBL-producing Enterobacterales, and newer agents such as meropenem/vaborbactam or ceftazidime/avibactam in carbapenem-resistant Enterobacterales pneumonia. Potential initial treatment options for pneumonia caused by MDR *P. aeruginosa* include antipseudomonal cephalosporins, carbapenems, fluoroquinolones or BL/BLIs, while colistin combination therapy is recommended for pneumonia due to MDR *A. baumannii.*^54^

### Complicated UTIs

Urinary tract infections (UTIs) represent the highest proportion of healthcare-acquired infections, at approximately 40%.^55^ Resistant Gram-negative bacteria are increasingly causing complicated UTIs (cUTIs), mainly due to the spread of ESBL-producing bacteria.^56^^,^^57^*E. coli* and other common Enterobacterales, including *Klebsiella and Pseudomonas* spp, are common causes of cUTIs.^57^^,^^58^ Scottish guidelines for treating UTIs include guidance for MDR organisms, recommending nitrofurantoin, pivmecillinam, trimethoprim or fosfomycin.^59^ The 2020 IDSA guidelines provide detailed recommendations for cUTI depending on the type of MDR, with separate recommendations for ESBL-producing Enterobacterales, CRE and *P. aeruginosa* with difficult-to-treat resistance (Tables 2 and 3).^35^ In severe UTIs, the following options are recommended by Muntean *et al*.^55^: cefepime, ceftazidime, imipenem, doripenem, meropenem and piperacillin/tazobactam. The latter also stress that empirical treatment must consider risk factors for resistant infections, namely duration of hospitalization, previous administration of antibiotics, and local resistance patterns.^55^

### Other clinical scenarios

Guidelines for the treatment of complicated intra-abdominal infections (cIAI) have been published by the World Society of Emergency Surgery.^60^ Piperacillin/tazobactam is the common treatment of choice in this indication, followed by a carbapenem. However, the use of piperacillin/tazobactam to treat infections caused by ESBL-producing Enterobacterales remains controversial, and it should be reserved for stable, rather than critically ill, patients.^60^ Ceftolozane/tazobactam or ceftazidime/avibactam are recommended, as part of a carbapenem-sparing strategy, in critically ill patients with hospital-acquired IAIs; however, these agents must be used in combination with metronidazole^60^ as they have limited or variable activity against anaerobic bacteria.^60–62^ In contrast, the *in vitro* anaerobic activity of meropenem/vaborbactam is similar to that of meropenem alone,^63^ and this agent is approved as monotherapy for treatment of adults with cIAI in Europe.^64^

## Main therapies currently used for MDR Gram-negative bacterial infections

### Older treatments

Older antimicrobials are still commonly used for treating Gram-negative infections, usually as combinations, especially where certain types of MBL-producing organisms are common, and for carbapenem-resistant *P. aeruginosa and A. baumannii*, although their use in infections caused by KPC-producing CRE is more questionable.^33^ These treatment combinations have not been studied in well-designed clinical studies, thus evidence supporting their use is based on data from retrospective studies, and pharmacokinetic and pharmacodynamic characteristics have been derived from empirical data. The safety profiles and related limitations of these agents are well known and often negatively impact patient outcomes, particularly when used to treat MDR infections. These agents include colistin, fosfomycin, tigecycline, aminoglycosides, piperacillin/tazobactam and high-dose carbapenems.

#### Colistin

The optimal use of colistin in MDR Gram-negative infections is subject to debate. Despite its association with nephrotoxicity and neurotoxicity, there has been a resurgence in its use because of the increasing prevalence of carbapenem-resistant species.^65^ In MDR Gram-negative nosocomial pneumonia, colistin is used in an effort to address the high morbidity and mortality rates in patients hospitalized for pneumonia.^54^ A recent position statement by the Clinical and Laboratory Standards Institute (CLSI) and European Committee on Antimicrobial Susceptibility Testing (EUCAST) noted that colistin needs to be used at a dose that achieves steady-state levels in excess of 2 mg/L, yet less than half of patients achieve this level of colistin exposure because of concerns about nephrotoxicity.^66^ In an Italian cross-sectional study assessing colistin use in high-risk adults (including those with recent hospitalization, and multiple comorbidities), colistin was given most often in combination with agents for MDR Gram-negative organisms, mainly for the targeted therapy of lower respiratory tract infections and BSIs caused by carbapenem-resistant organisms.^67^ However, 30 day mortality in patients with pneumonia (mostly caused by *Klebsiella*) who were treated with colistin was three-fold higher than in those treated with ceftazidime/avibactam (32% versus 9%; absolute difference 23%, 95% CI 9%–35%; *P = *0.001); patients in both groups received add-on anti-CRE agents.^68^ Furthermore, resistance to colistin is now emerging, as evidenced by a highly virulent strain of *Escherichia coli* found to be resistant to colistin via the *mcr-1* gene.^65^ The same *E. coli* strain is resistant to numerous other antibiotics, including most BLs and all non-BLs.^65^ Furthermore, evidence suggests that colistin, alone or in combination, has no impact on clinical outcomes or mortality,^69^ and is often associated with nephrotoxicity in severely ill patients.^70^

#### Fosfomycin

Fosfomycin was discovered in the 1960s and has been used for many years, particularly in the treatment of UTIs.^71^ It has a unique mechanism of action, inhibiting UDP-GlcNAc enolpyruvyl transferase, the first step of the synthesis of bacterial cell walls, rendering it useful in the treatment of resistant Gram-negative infections.^71^ Indeed, resistance is the driver of the recent increase in its use.^72^ Resistance to fosfomycin itself is most commonly via amino acid replacement or peptidoglycan recycling in the formation of the bacterial wall, and cross-resistance is uncommon.^73^ Fosfomycin is particularly active against *E. coli* as well as some carbapenem-resistant bacteria.^71^ In the ZEUS study, which compared injectable fosfomycin with piperacillin/tazobactam in patients with cUTIs caused mostly by *E. coli*, fosfomycin was non-inferior in the primary outcome of clinical cure and microbial eradication.^74^*E. coli* eradication was 100% with fosfomycin.^74^ Hypokalaemia was more common in the fosfomycin group.^74^ In fact, intravenous (IV) fosfomycin is known to be associated with sodium overload and hypokalaemia, and therefore, administration of potassium supplements is recommended.^75^ Furthermore, because fosfomycin is eliminated mostly by the kidneys, it should be administered with caution in patients with renal impairment and dose adjustments may be necessary.^75^ A 2008 review evaluated numerous case studies and clinical trials of fosfomycin against a variety of non-UTI or gastrointestinal infections involving Gram-negative bacilli (most commonly *P. aeruginosa*), usually in combination with other antimicrobials.^76^ The overall results showed a cure rate of 81.1%, indicating that the usefulness of fosfomycin may be extended beyond UTIs.^76^ Fosfomycin is generally well tolerated.^76^

#### Tigecycline

While active *in vitro* against carbapenem-resistant Enterobacterales and carbapenem-resistant *A. baumannii*, but not carbapenem-resistant *P. aeruginosa*,^77^ tigecycline is generally used in combination with other agents.^32^^,^^78^ It is recommended for use in MDR skin and soft tissue infections (SSTIs) and abdominal infections, and in combination with other agents for hospital-acquired respiratory infections,^19^ although not for VAP.^79^ While one study found high-dose tigecycline to be the only independent predictor of clinical cure in critically ill patients with VAP and MDR bacterial infections (carbapenem-resistant *A. baumannii* or carbapenem-resistant *K. pneumoniae*),^80^ a meta-analysis of clinical studies found an increased risk of mortality with tigecycline versus active comparators,^81^ which led to a Black Box warning and change in the US labelling against its use in VAP.^79^ There is evidence of *A. baumannii* resistance to tigecycline via overexpression of the AdeABC efflux pump,^82^ and of breakthrough infection; furthermore, tigecycline may not achieve the tissue levels necessary to treat pneumonia, meaning high-dose therapy is often needed.^83^ Therefore, high-dose tigecycline-based combinations should be reserved for critically ill patients with carbapenem-resistant Enterobacterales infections and limited treatment options.^84^

#### Aminoglycosides

Aminoglycosides have been used for many decades to treat Gram-negative nosocomial pneumonia.^85^ and more recently have been used to treat infections caused by carbapenem-resistant Gram-negative bacteria.^32^ Traditionally used in combination, aminoglycosides such as amikacin, gentamicin and tobramycin are used as monotherapy in UTIs only,^78^ where they demonstrate considerable effectiveness. In carbapenem-resistant Enterobacterales-associated conditions other than UTIs, they are associated with unacceptably high mortality (up to 80%) when given alone.^78^ They are used in cases of polymyxin resistance, but aminoglycosides are susceptible to several resistance mechanisms, including reduced uptake, target modification through mutation, and enzymatic inactivation.^32^ Furthermore, when given in combination with IV BLs, IV aminoglycosides increase the risk of nephrotoxicity compared with a BL alone in patients with VAP.^7^ Inhaled amikacin initially showed promise, but the recent IASIS and INHALE studies have shown no clinical benefit in adding inhaled amikacin to IV standard of care for VAP.^86^

Of note, aminoglycosides are known to cause nephrotoxicity.^32^ In addition, they have decreased activity at lower pH of airway linings and the concentrations of aminoglycosides detected in the lung tissues may not be sufficient to effectively treat VAP.^32^^,^^87^

#### Piperacillin/tazobactam

The BL/BLI piperacillin/tazobactam is a broad-spectrum antibiotic with activity against multiple Gram-negative pathogens and is one of the few agents that is active against *Pseudomonas* spp.^88^^,^^89^ In patients with *E. coli* or *K. pneumoniae* BSI and ceftriaxone resistance, piperacillin/tazobactam showed no benefit over meropenem in terms of 30 day mortality.^47^ The ZEUS study found piperacillin/tazobactam to be somewhat less effective than fosfomycin in patients with UTIs.^74^ However, it was noted that the dose of the former may have been sub-optimal in that study.^72^

In many critically ill patients, as well as patients with mild or moderate renal impairment, the typical dose of piperacillin/tazobactam (4.5 mg three times daily) is insufficient to achieve effective bactericidal concentrations, and dose adjustments may be required.^90^

### Newer treatment options

#### Ceftazidime/avibactam

Ceftazidime/avibactam is a novel combination of the third-generation cephalosporin ceftazidime and the BLI avibactam, with indications including HAP and VAP.^91^ Ceftazidime/avibactam was approved in the USA in 2015 for the treatment of cIAIs (in combination with metronidazole) and cUTIs, including pyelonephritis, in patients aged ≥18 years.^92^ In addition to these indications, ceftazidime/avibactam was approved in Europe in 2016 for the treatment of HAP, including VAP^93^ and, as of June 2020, for the treatment of bacteraemia associated with, or suspected to be associated with any of the above infections.^93^ The International Network For Optimal Resistance Monitoring global surveillance programme (2012–15) demonstrated 99.4% susceptibility to ceftazidime/avibactam for all Enterobacterales isolates and 98.5% susceptibility for meropenem-non-susceptible, MBL-negative isolates.^78^ In a study of antimicrobial activity against carbapenem-resistant Enterobacterales isolated from ICUs in Taiwan, ceftazidime/avibactam demonstrated susceptibility rates of 99% for *E. coli*, 100% for *K. pneumoniae* and 91% for *P. aeruginosa.*^94^ Ceftazidime/avibactam given as monotherapy or in combination with other agents was superior to other treatment regimens, including carbapenem plus aminoglycoside, colistin and other regimens, against carbapenem-resistant *K. pneumoniae* bacteraemia.^95^ In patients with KPC-producing *K. pneumoniae* infections, ceftazidime/avibactam proved to be a reasonable alternative treatment option to colistin, and was associated with a lower risk of nephrotoxicity.^68^ In a retrospective study that included 138 patients with KPC-producing *K. pneumoniae* infections, ceftazidime/avibactam was effective as salvage therapy following first-line treatment with other antimicrobials.^96^ Given ceftazidime’s inhibitory profile against OXA-48-like enzymes and its stability in the presence of these hydrolysing enzymes, ceftazidime/avibactam is the preferred agent for the treatment of infections caused by OXA-48-producing carbapenem-resistant Enterobacterales.^97^

The REPROVE study confirmed that ceftazidime/avibactam is non-inferior to meropenem in HAP, including VAP (clinical cure rate 68.8% versus 73.0%).^98^ However, the cure rate with ceftazidime/avibactam was lower than expected based on preclinical pharmacokinetic and pharmacodynamic data.^99^ Moreover, cases of *K. pneumoniae* resistance emerged during a clinical trial with ceftazidime/avibactam; the resistance was found to be caused by plasmid-borne mutations (D179Y/T243M) at position 243 in the *bla*_KPC-3_ genes.^100^

A key, currently unaddressed question is the optimal therapeutic regimen of ceftazidime/avibactam for carbapenem-resistant Enterobacterales (i.e. whether it should be given as monotherapy or in combination with other agents). Pharmacokinetic/pharmacodynamic optimization by administering prolonged (>2 h) or continuous infusions could be a key strategy to prevent treatment failure with ceftazidime/avibactam and subsequent development of resistance,^101^ but this approach has not yet received regulatory approval.

#### Meropenem/vaborbactam

Meropenem/vaborbactam has been approved in the USA for the treatment of patients aged 18 years and older with cUTIs, including pyelonephritis, since 2017.^102^ In Europe, meropenem/vaborbactam was approved in 2018 for the treatment of adults with cUTIs, including pyelonephritis, cIAIs and HAP, including VAP.^64^ Meropenem/vaborbactam is also indicated for the treatment of patients with bacteraemia that occurs in association with, or suspected to be associated with, any of the above infections and for treatment of infections due to bacterial organisms in adults with limited treatment options.^64^ The combination of the well-known, broad-spectrum carbapenem meropenem with vaborbactam, a first-in-class boronic acid inhibitor of class A and class C β-lactamases, had excellent *in vitro* activity against KPC-producing Enterobacterales isolates from around the world collected in 2014 and 2015 (99.0% susceptibility).^103^ Meropenem/vaborbactam demonstrated marked activity against Enterobacterales strains producing KPC carbapenemases, with less but still notable activity against those that produce MBLs or OXA-48-like enzymes.^98^^,^^104^^,^^105^ It is administered as a high dose prolonged infusion (2 g meropenem, 2 g vaborbactam over 3 h) every 8 h to optimize pharmacokinetic/pharmacodynamic exposures, resulting in enhanced bacterial killing and EUCAST species-related breakpoints for Enterobacterales and *P. aeruginosa* of susceptible ≤8 mg/L and resistant >8 mg/L.^106^*P. aeruginosa* is considered a clinically relevant pathogen for meropenem/vaborbactam in Europe but not in the US.

Meropenem/vaborbactam was approved in the USA in 2017 for the treatment of cUTIs and acute pyelonephritis^91^ based on the results of the TANGO 1 study, which showed non-inferiority of meropenem/vaborbactam to piperacillin/tazobactam.^107^ In the Phase III TANGO 2 study, meropenem/vaborbactam as monotherapy was compared with best available therapy in a representative group of patients with CRE infections (bacteraemia 36.0%, cUTI/acute pyelonephritis 45.3%, HAP/VAP 9.3% and cIAIs 9.3%), including those with multiple comorbidities, compromised immune systems and moderate-to-severe renal impairment.^108^ Meropenem/vaborbactam was associated with significantly higher rates of clinical cure than best available therapy [65.6% (21/32) versus 33.3% (5/15); difference, 32.3%; 95% CI 3.3%–61.3%, *P = *0.03] at the end of treatment.^108^ Meropenem/vaborbactam was also associated with numerically lower 28 day mortality (15.6% versus 33.3%) and fewer renal-related adverse events (4.0% versus 24.0%) compared with best available therapy. The study was concluded early in favour of meropenem/vaborbactam based on a risk/benefit analysis by the Data Safety Monitoring Board.^108^ Early real-world experience with meropenem/vaborbactam further supports the effectiveness of meropenem/vaborbactam demonstrated in clinical studies, showing that the combination was able to achieve clinical success in 70% of severely ill patients with Gram-negative CRE infections, including nosocomial pneumonia, cUTI, intra-abdominal and SSTIs.^109^

#### Ceftolozane/tazobactam

Ceftolozane/tazobactam is another BL/BLI combination.^91^ It was approved in the USA in 2014 for the treatment of cIAIs (in combination with metronidazole) and cUTIs, including pyelonephritis.^110^ In Europe, ceftolozane/tazobactam was approved in 2015 for the treatment of cIAIs, acute pyelonephritis, cUTIs and HAP, including VAP.^111^ Unlike some other cephalosporins, it is active against AmpC β-lactamases, especially *P. aeruginosa*.^91^ In the ASPECT-NP study, ceftolozane/tazobactam was non-inferior to meropenem for treating Gram-negative nosocomial VAP.^112^ However, it should be noted that in this study, ceftolozane/tazobactam was administered at twice its first approval’s recommended dose (3 g versus 1.5 g every 8 h).^112^ Cure rates with ceftolozane/tazobactam have been found to be lower in patients with renal impairment, so dose adjustment may be required in patients with impaired renal function^91^ UK clinical practice guidelines recommend ceftolozane/tazobactam for the treatment of cUTI caused by resistant Gram-negative infections.^58^

In cIAIs, overall clinical cure rates were 83.0% with ceftolozane/tazobactam plus metronidazole and 87.3% with meropenem.^113^ When stratified by pathogen, the clinical cure rates for all patients with ESBL-producing Enterobacterales were 95.8% and 88.5%, respectively.^113^

It may be a useful treatment option for severe infections caused by carbapenem-resistant *P. aeruginosa* provided susceptibility is confirmed. A real-world study of patients infected with carbapenem-resistant *P. aeruginosa* reported a clinical cure rate of 74%.^114^

#### Imipenem/relebactam

Relebactam is a novel, IV class A and C BLI which, when combined with imipenem, restores the latter’s activity against the KPC-producing CREs, *K. pneumoniae and P. aeruginosa*, but not *A. baumannii.*^33^^,^^91^ Similarly, imipenem/relebactam has shown *in vitro* activity against KPC-producing Enterobacterales and MDR *P. aeruginosa*, but was not active against *A. baumannii* clinical isolates.^115^ In 2019, imipenem/relebactam was approved in the USA for the treatment of cUTIs, including pyelonephritis, and cIAIs in patients aged ≥18 years with limited or no alternative treatment options.^116^ In Europe, it was approved in 2020 for the treatment of infections caused by aerobic Gram-negative organisms in adults with limited treatment options.^117^ Imipenem/relebactam has been investigated in the treatment of imipenem-resistant HAP, VAP, cIAI and cUTI.^91^ In cUTI, imipenem/cilastatin + relebactam was non-inferior to imipenem/cilastatin alone, with over 95% of patients treated with either imipenem/cilastatin + relebactam 250 mg, imipenem/cilastatin + relebactam 125 mg or imipenem/cilastatin + placebo having favourable microbiological responses.^118^ RESTORE-IMI 1, conducted in patients with HAP/VAP, cIAI or cUTI caused by imipenem-resistant Enterobacterales, reported favourable responses in 71% of patients receiving imipenem/relebactam and 70% of those receiving colistin + imipenem overall; favourable responses were not found in patients with cIAI.^119^

#### Cefoperazone/sulbactam

Cefoperazone/sulbactam was found to have *in vitro* activity against 91.6% of Enterobacterales according to recent data published on behalf of the SENTRY antimicrobial surveillance programme, meaning it is one of the most active compounds *in vitro.*^120^ Susceptibility rates varied by region, ranging from 94.4% in Western Europe to 82.0% in Eastern Europe.^120^ In a study comparing cefoperazone/sulbactam with tigecycline for BSI due to carbapenem-resistant *A. baumannii*, 28 day mortality was significantly higher with tigecycline.^121^ In patients with BSI due to ESBL-producing Enterobacterales, there were no statistically significant differences between patients treated with cefoperazone/sulbactam and those treated with a carbapenem in terms of success rates (70.6% versus 73.9%, odds ratio 0.847, *P = *0.761), sepsis-related mortality or 14 day mortality.^122^ In HAP or healthcare-associated pneumonia, cefoperazone/sulbactam demonstrated non-inferiority to cefepime, with a similar number of patients defined as cured at the end of the study.^123^ Cefoperazone/sulbactam is currently approved in some European countries (Bulgaria, Czech Republic, Italy, Lithuania, Poland, and Slovakia), but not in the USA.

#### Eravacycline

Eravacycline is a tetracycline antibiotic that is effective *in vitro* against many microorganisms that are resistant to other tetracyclines, including MDR *Acinetobacter* spp. and ESBL-producing Enterobacterales spp.^124^^,^^125^ In 2018, eravacycline was approved in the USA and Europe for the treatment of cIAIs in patients aged ≥18 years.^126^^,^^127^ Two randomized, double-blind studies [Investigating Gram-Negative Infections Treated with Eravacycline (IGNITE)] demonstrated that, in terms of clinical cure rates, eravacycline was non-inferior to ertapenem (IGNITE1) and to meropenem (IGNITE4) in patients with cIAIs.^128^^,^^129^ Eravacycline is generally well tolerated; the most common adverse events are nausea, vomiting and infusion site reactions.^126^ Eravacycline is expected to provide a valuable therapeutic option for patients who cannot tolerate β-lactams or fluoroquinolones, and may help reduce the use of quinolones, carbapenems and BLIs.^125^

#### Plazomicin

The novel semisynthetic aminoglycoside plazomicin is active against Enterobacterales, but is less active against non-fermenting Gram-negative bacteria,^91^ due to its vulnerability to ribosomal ribonucleic acid methyltransferases.^78^ Plazomicin is currently approved by the US Food and Drug Administration for the treatment of cUTI; however, the application for marketing authorization for plazomicin in Europe was withdrawn in 2020.^91^^,^^130^ Plazomicin was evaluated in patients with serious carbapenem-resistant Enterobacterales infections in the CARE study, which, although it was terminated early because of low study enrolment, found that plazomicin was associated with reduced all-cause mortality at 28 days compared with colistin (24% versus 50% of patients, 95% CI –55 to 6).^131^

Plazomicin was found to be more active *in vitro* than traditional aminoglycosides.^132^ In the EPIC study, it was non-inferior to meropenem in the treatment of patients with cUTI.^133^ These results, taken together with the encouraging results from the CARE study,^131^ suggest that plazomicin may have an important role in the management of carbapenem-resistant Enterobacterales infections, particularly cUTIs.^133^

#### Cefiderocol

The novel siderophore cephalosporin cefiderocol is active *in vitro* against a variety of Ambler class A, C and D β-lactamases, and it is the first agent with activity versus class B β-lactamases. This confers activity against MDR Gram-negative bacilli, including MDR Enterobacterales, *P. aeruginosa, A. baumannii and Stenotrophomonas maltophilia*, while possessing a safety and tolerability profile similar to that of other cephalosporins.^91^ Cefiderocol was assessed in the Phase III study, CREDIBLE-CR, to compare its effectiveness with that of best available therapy in patients with CRE Gram-negative pneumonia, cUTI or BSI/sepsis.^134^ The results showed respective clinical cure rates for cefiderocol versus best available therapy of 50.0% versus 52.6%, 70.6% versus 60.0%, and 43.5% versus 42.9%.^135^ However, all-cause mortality was higher in patients who received cefiderocol than in those who received best available therapy.^136^^,^^137^ Cefiderocol was approved in the USA in 2019 and in Europe in 2020 for the treatment of infections, including pyelonephritis, caused by Gram-negative microorganisms in patients aged ≥18 years; however, the indication is limited to patients who have limited or no alternative treatment options.^136^^,^^137^

### Potential future antibiotics

#### Cefepime/zidebactam

Cefepime/zidebactam combines the diazabicyclooctane (DBO) zidebactam, a second-generation BLI, with the broad-spectrum cephalosporin cefepime. *In vitro*, zidebactam demonstrated higher potency than avibactam or relebactam against class C β-lactamases.^29^ When available, cefepime/zidebactam could provide a much-needed antibacterial agent in the fight against MDR Gram-negative pathogens. A study assessing the safety, tolerability and pharmacokinetics of IV cefepime/zidebactam in healthy volunteers has been completed (NCT02707107).^138^

#### Meropenem/nacubactam

Nacubactam is another DBO BLI. Combined with meropenem, it has demonstrated *in vivo* effectiveness against carbapenem-resistant *K. pneumoniae, E. coli* and AmpC-depressed *P. aeruginosa.*^139^ A study examining the intrapulmonary penetration of nacubactam combined with meropenem in healthy volunteers has recently been completed (NCT03182504).^140^

#### Cefepime/enmetazobactam

Another potential agent for the treatment of ESBL-expressing Enterobacterales is cefepime/enmetazobactam. Enmetazobactam has been shown to restore the activity of cefepime and piperacillin against selected ESBL-producing strains more potently than tazobactam.^141^*In vitro*, cefepime/enmetazobactam was as effective as meropenem and imipenem against the same ESBL-producing strains.^141^

### Long-term view: new targets for antimicrobials

#### Phages

Bacteriophages are viruses that specifically target bacteria by disrupting almost all bacterial cellular processes.^82^ They have several advantages over conventional antibiotics in that they are highly species- or strain-specific, and as such are less likely to cause dysbacteriosis and secondary infections.^82^ However, they are also susceptible to bacterial resistance and may themselves contribute to resistance by acting as vehicles for the acquisition, maintenance and spread of antibiotic resistance genes.^82^

#### Odilorhabdins

Odilorhabdins are a new class of modified peptide antibiotics produced by enzymes encoded in an identified non-ribosomal peptide synthetase gene cluster present in the genome of *Xenorhabdus nematophila.*^142^ Odilorhabdins have a unique mechanism of action in that they target a site on the small bacterial ribosomal subunit not targeted by any known ribosome-targeting antibiotic. NOSO-95179 is a synthetic version of naturally occurring odilorhabdin and has demonstrated activity against a wide range of Gram-negative pathogens including *K. pneumoniae, E. coli* and difficult-to-treat CRE.^142^

## Major considerations for future management of resistant infections

Important broad principles for the future management of antimicrobial resistance include improvement in diagnostic and prescribing practices, reduction of antimicrobial use in agriculture, development of new antimicrobials, antimicrobial stewardship programmes, more equitable access to medications, and improved surveillance and infection control programmes.^25^ This list is extensive and challenging, but implementing these principles is fundamental to the continuing management of antimicrobial resistance worldwide.

One of the most important anticipated developments in the management of antibiotic-resistant infections is the introduction of novel diagnostic tools.^143^ At present, empirical treatment continues to be the most common approach, but contributes to the misuse of antibiotics and, therefore, the spread of antibiotic resistance. Furthermore, traditional growth-based techniques for assessing antibiotic susceptibility are time-consuming and require pure cultures. On the other hand, novel diagnostic techniques that rely on nucleic acid amplification, nucleic acid hybridization or immunodiagnostic methods can be applied to non-purified samples. Such techniques promise to provide rapid, point-of-care diagnosis and antibiotic susceptibility testing. This is expected to reduce treatment delays and enable the shift to evidence-based treatment, thereby preventing unnecessary use of antibiotics and the spread of antibiotic resistance. They may also reduce the overall cost of treatment by eliminating the need to purify and grow cultures.^143^

Antimicrobial stewardship programmes must include leadership commitment by infection experts, collaboration between stewardship teams and primary care physicians, and treatment algorithms for appropriate dosing and de-escalation of antibiotics according to culture and susceptibility results.^9^ With the steady increase in carbapenem resistance, and the continued high use of these agents, carbapenems must be utilized appropriately, particularly in hospitals.^9^ The WHO priority list of pathogens^144^ provides a framework to continue development of new antimicrobial agents and combinations of new and older agents, including some of those discussed here. However, many newer agents are not yet ready for clinical use despite showing high levels of activity *in vitro*, and bringing them to the approval stage takes time.^145^ It is not always possible to conduct randomized controlled studies involving the required number of patients in a timely manner, given the relatively small number of patients with certain MDR infections.^34^ In particular, in order to determine the place of new agents in treatment algorithms, these agents must undergo comparative studies with established agents.^34^ Newer agents may be more effective and better tolerated, but are also more costly; costs can be reduced through de-escalation protocols, when applicable, and these factors also need to be considered in any AMS programme.^34^ There is also a need for government-based financial incentives to promote the research and development of new antimicrobial agents, which may help combat MDR infections.^146^^,^^147^ Reimbursement decisions should consider the unique properties of novel antimicrobial agents in order to improve their market use and create incentives for pharmaceutical development of these agents.^147^

Geographic differences in the rates of resistance highlight the need to adapt empirical treatment to local epidemiology, patient risk stratification and local stewardship protocols. Rapid diagnostics are needed to guide management, including targeting treatments appropriately and rapid de-escalation from broad-spectrum agents when possible.

## Conclusions

The WHO priority list of pathogens^144^ provides an impetus and a framework to continue development of new antimicrobials and combinations of new and older agents in order to combat the increase in MDR Gram-negative pathogens. The success of new antimicrobial agents depends upon increased efforts to promote research and development by governments around the world, as well as robust antimicrobial stewardship programmes and detailed local knowledge of resistance. There is also a need to put procedures in place to reduce inappropriate prescribing and misuse of antimicrobial agents in agriculture in order to ensure the success of future antimicrobial treatment.

## Transparency declarations

Outside the submitted work, M.B. has participated in advisory boards and/or received speaker honoraria and/or has received study grants from Angelini, Astellas, Bayer, BioMérieux, Cidara, Gilead, Menarini, MSD, Pfizer and Shionogi. J.G. has received speaker honoraria and participated in Advisory Boards from Menarini, Pfizer, MSD, Navriba, Paratek, Pfizer, Shionogi, and VenatoRx.

Marion Barnett prepared a first draft of this manuscript on behalf of Springer Healthcare Communications. This medical writing assistance was funded by A. Menarini Farmaceutica Internazionale.

This paper was published as part of a Supplement sponsored and financially supported by A. Menarini Farmaceutica Internazionale.
